# The Psychological Evaluation of Patients with Chronic Pain: a Review of BHI 2 Clinical and Forensic Interpretive Considerations

**DOI:** 10.1007/s12207-014-9206-y

**Published:** 2014-11-06

**Authors:** Daniel Bruns, John Mark Disorbio

**Affiliations:** 1 Health Psychology Associates, 1610 29th Avenue Place Suite 200, Greeley, CO 80634 USA; 2 113 Blue Grouse Road, Evergreen, CO 80634 USA

**Keywords:** Chronic pain, Psychological assessment, Psychological evaluation, Psychological screen, Forensic, Litigation, Battery for Health Improvement 2, Brief Battery for Health Improvement 2, BHI 2, BBHI 2, Presurgical, Treatment outcome, Standardized test, Opioid risk, Health psychology, Rehabilitation, Risk, Suicide, Violence, Litigiousness

## Abstract

Pain is the most common reason why patients see a physician. Within the USA, it has been estimated that at least 116 million US adults suffer from chronic pain, with an estimated annual national economic cost of $560–635 billion. While pain is in part a sensory process, like sight, touch, or smell, pain is also in part an emotional experience, like depression, anxiety, or anger. Thus, chronic pain is arguably the quintessential biopsychosocial condition. Due to the overwhelming evidence of the biopsychosocial nature of pain and the value of psychological assessments, the majority of chronic pain guidelines recommend a psychological evaluation as an integral part of the diagnostic workup. One biopsychosocial inventory designed for the assessment of patients with chronic pain is the Battery for Health Improvement 2 (BHI 2). The BHI 2 is a standardized psychometric measure, with three validity measures, 16 clinical scales, and a multidimensional assessment of pain. This article will review how the BHI 2 was developed, BHI 2 concepts, validation research, and an overview of the description and interpretation of its scales. Like all measures, the BHI 2 has strengths and weaknesses of which the forensic psychologist should be aware, and particular purposes for which it is best suited. Guided by that knowledge, the BHI 2 can play a useful role in the forensic psychologist’s toolbox.

Pain is the most common reason why patients see a physician: *Something hurts* (Centers for Disease Control and Prevention 2010; National Center for Health Statistics 1992). Thus, the presence of pain is a primary driving force that underlies the demand for health care. Within the USA, it has been estimated that at least 116 million US adults suffer from chronic pain, with an estimated annual national economic cost of $560–635 billion (Institute of Medicine 2011). Chronic pain is also closely associated with disability (Ehde et al. 2003; Zale et al. 2013). In 2012, 10.8 million persons were receiving Social Security Disability Income (SSDI), at the cost of $128 billion annually, not including the associated costs of Medicare (Congressional Budget Office 2012). Of SSDI recipients in 2011, 44 % were prescribed opioid pain relievers (Morden et al. 2014).

A recent report from the Institute of Medicine studied the problem of pain in the USA. It concluded that pain has biological, psychological, and social components, and effective treatments for pain must address all three of these components. This report stated that “effective pain management is a moral imperative, a professional responsibility, and the duty of people in the healing professions” (Institute of Medicine 2011; p. S-3). The International Association for the Study of Pain has affirmed the biopsychosocial nature of pain and concluded that pain has a dual nature. While pain is in part a sensory process, like sight, touch, or smell, pain is also in part an emotional experience, like depression, anxiety, or anger (Merskey and Bogduk 1994). At the neurophysiological level, the experience of pain is inextricably linked with physiological arousal, mood, memory, and cognition (Apkarian et al. 2009; Melzack 2001). Thus, chronic pain represents the quintessential biopsychosocial condition.

One of the challenges of treating patients with chronic pain is that pain leads to increased demand for opioid medication to suppress the pain, and surgical interventions to “fix” the pain. Unfortunately, due to pain’s complex nature, iatrogenic complications are common. One study examined the number of unintentional deaths in the USA from prescription opioid pain medications and found that it exceeds the number of deaths due to cocaine and heroin combined (Centers for Disease Control and Prevention 2012). Another study examining the effects of surgical treatments for patients with spinal pain found that while an objectively successful fusion occurred in 84 % of lumbar fusion patients, nearly half were dissatisfied with their outcome, and many were totally disabled at follow-up (LaCaille et al. 2005). Because pain treatments such as these can lead to iatrogenic complications, these treatments can be both costly and counter-productive.

The study by LaCaille and colleagues above illustrates an important conclusion of another study. In many cases, orthopedic surgeries for chronic pain are performed when the primary outcome goals are to change behavior: to induce the patient to say, “My pain is much better,” to say, “I don’t need opioids anymore,” to report satisfaction with health care, or to return to work (Bruns and Disorbio 2009). While medical imaging techniques are helpful for making objective diagnoses, these imaging techniques were not designed to assess feelings or predict behavior. Consequently, pain self-reports cannot be replaced by neuroimaging or other technologies (Robinson et al. 2013). Given the prominent psychological component of chronic pain, the advice of Hippocrates is especially apt: “It is more important to know what sort of person has a disease, than to know what sort of disease a person has” (Hippocrates, 400 BCE). Thus, since chronic pain is known to be a complex, biopsychosocial condition, a prerequisite of effective pain treatment is accurate assessment of not only the medical aspects of pain but also the psychosocial aspects as well.

Psychological interventions for chronic pain have been shown to be safe and effective treatments for pain (Hoffman et al. 2007) but are generally underutilized (Robbins et al. 2003). Studies have shown that psychological treatments combined with exercise can produce improvements in functioning that equal those of surgery for back pain (Brox et al. 2010; Chou et al. 2009). The economic benefits of a treatment model that integrated psychological services was tested in a 15-year longitudinal study of 29 million patients, which provided evidence that a biopsychosocial model for treating pain and injury provided better care at less cost (Bruns et al. 2012b). This model relied heavily on psychological assessments for treatment planning.

An extensive review of the evidence determined that psychological tests are the scientific equal of medical tests (G. J. Meyer et al. 2001) and can sometimes exceed the ability of medical tests to predict the outcome of medical treatments for pain (Carragee et al. 2005; Carragee et al. 2004). Due to the overwhelming evidence for the biopsychosocial nature of pain and the value of psychological assessments, the majority of chronic pain guidelines recommend a psychological evaluation as an integral part of the diagnostic workup (Bruns in press). These guidelines create a mandate for both clinical and forensic psychological evaluations of chronic pain.

Psychological assessments for medical patients serve a number of purposes. These include providing an accurate means of describing a medical patient’s mental status, medical symptoms, traits, attitudes, abilities, and the patient’s perception of the social environment. This in turn can facilitate making determinations about how to diagnose or classify the patient, plan interventions, predict outcome, and measure change (Bruns in press; Bruns and Disorbio 2013; Turner et al. 2001). One biopsychosocial inventory designed for the assessment of patients with chronic pain is the Battery for Health Improvement 2 (BHI 2) (Bruns and Disorbio 2003). The BHI 2 is a standardized psychometric measure, a primary purpose of which is to perform clinical and forensic evaluations of patients with pain and injury. This article will provide an overview of how the BHI 2 was developed, BHI 2 interpretive concepts, and the nature and validation of its scales.

## Overview of the BHI 2

The goal of the BHI 2 development was to create a comprehensive biopsychosocial inventory to assess medical patients with pain and/or injury specifically, and somatic symptom disorders more generally. Consequently, the BHI 2 is best conceptualized not as a psychological inventory, but rather as a biopsychosocial inventory.

A brief review of the BHI 2’s development and validation process (Bruns and Disorbio 2003) is as follows:

1. The BHI 2 originated with a paradigm called the vortex model, which is a graphical representation of the biopsychosocial model as it pertains to the onset of injury, illness, chronic pain, and intractable biopsychosocial disorders (Fig. 1). This model attempted to organize what was known about how patients respond to health challenges, and why some patients get into a “downward spiral” of worsening symptoms. The vortex model served as a guide for BHI 2 development, and as a conceptual paradigm for performing a biopsychosocial evaluation in the clinical setting.

**Fig. 1:**
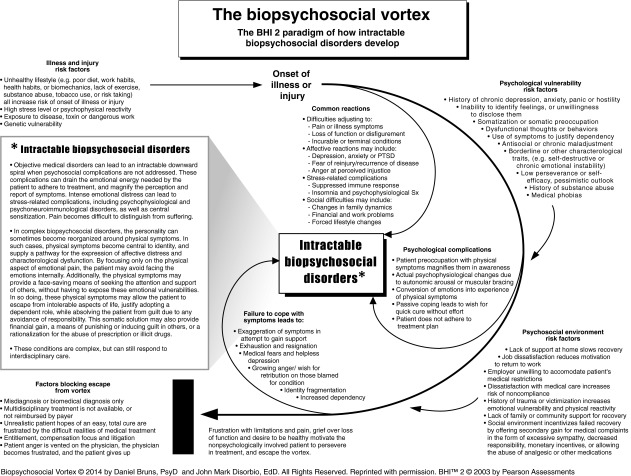
The Vortex Paradigm from which the BHI 2 was developed
2. Development of the BHI 2 began when, based on this biopsychosocial paradigm, over 1,100 items were generated, of which 600 were selected for empirical assessment.
3. The 600 selected items were administered to 2,507 subjects gathered from 106 sites in 36 US states, along with a number of other measures. Information from treating providers was also gathered.
4. From the overall sample, patient and community development groups were identified, and these were used to explore the psychometrics of the prototypical BHI 2 scales.
5. Later, patient and community normative groups were also developed and were stratified to match US Census data for age, gender, race, and education. These two norm groups serve as estimates of the responses of the average American patient with pain or injury, and the average American community member.
6. Eight other reference groups were also identified. From the patient norm group, subgroups for chronic pain, head injury/headache pain, neck pain, upper extremity pain, low back pain, and lower extremity pain were identified. Additionally, fake good and fake bad groups were also obtained.
7. The scales to develop were identified through a review of the literature and were represented in the vortex paradigm. The BHI 2’s scales are organized in accordance with the biopsychosocial model, and the content of the scales and items were developed to represent the various aspects of the paradigm.
8. Items were assigned to the 18 BHI 2 scales based on the appropriateness of the item content, and the ability of the item to differentiate one group of subjects from another, item to scale correlations, item to criteria correlations, and resultant scale to criteria correlations.
9. The BHI 2 development process produced three validity measures (Validity Items (random responding), Self-Disclosure, and Defensiveness), “biological” scales assessing medical symptoms (Somatic Complaints, Pain Complaints, Functional Complaints, and Muscular Bracing), psychological measures of affect and characterological dysfunction  (Depression, Anxiety, Hostility, Borderline, Symptom Dependency, Chronic Maladjustment, Substance Abuse, and Perseverance), and measures of the patient’s social environment (Family Dysfunction, Doctor Dissatisfaction, Survivor of Violence, and Job Dissatisfaction). At cross-validation, the mean test-retest reliability and Cronbach’s alpha of the BHI 2 scales were .93 and .84, respectively.
10. The development process also yielded 40 content-based subscales. Items were assigned to these subscales based on the appropriateness of the item content as determined by the opinion of a panel of 12 expert judges. The description of these scales goes beyond the scope of this paper. However, the content validity established by this method supported the content validity of the parent scale of which the subscale is a part. The mean test-retest reliability, Cronbach’s alpha, and interjudge agreement regarding item content of the BHI 2 content-based subscales were .88, .69, and .92, respectively.
11. Following the completion of the test development process, the BHI 2 has been the subject of numerous peer-reviewed studies about various matters of clinical interest.
12. Because the BHI 2 scales were validated in different ways, validity is a more complex topic and will be discussed on a scale-by-scale basis below.

Along with tests such as the Minnesota Multiphasic Personality Inventory-2-RF (Ben-Porath and Tellegen 2011), the Millon Clinical Multiaxial Inventory III (Millon et al. 1997), and the Millon Behavioral Medicine Diagnostic (Millon et al. 2001), the BHI 2 has been listed as a commonly used test for patients with chronic pain in multiple medical treatment guidelines (American College of Occupational and Environmental Medicine 2008; California Division of Workers' Compensation 2009; Colorado Division of Workers' Compensation 2012; Oklahoma Physician Advisory Committee 2007; Work Loss Data Institute 2009), has been integrated into clinical protocols (Bruns and Disorbio 2013), and was favorably peer reviewed by the Buros Center for Testing (Vitelli 2007). This review concluded that the BHI 2 could benefit from further studies about validity assessment and malingering, and longitudinal studies about medical treatment outcome. Despite these weaknesses, the review concluded that “the reliability and validity research of the BHI 2 demonstrates that it is one of the best instruments available for assessing the broad range of treatment needs in clinical populations” (Vitelli 2007; p. 75).

At the time of this writing, the BHI 2 or Brief BHI 2 (BBHI 2) has been accepted as evidence in several US federal court cases (Chambers v. Astrue 2013; Cowgar v. Commissioner Of Social Security Administration 2008; Cowger v. Astrue 2008; Davis v. Astrue 2009; Lewis v. Astrue 2012; Webb v. Astrue 2009). The short version of the BHI 2, the Brief Battery for Health Improvement 2 (BBHI 2), has been accepted as evidence in one US federal case (McGuire v. Astrue 2008). Although the BHI 2 and BBHI 2 have been accepted as evidence in federal courts, there have been no specific judicial rulings pertaining to the tests themselves. However, in one case, one psychologist’s testimony was determined to be more persuasive than that of a second psychologist due to the fact that the first psychologist had performed validity testing, which included the BHI 2 scales (*Webb v. Astrue*). In two other cases, testimony was given that the BHI 2 “is a more sophisticated test” than the Beck Depression Inventory (*Cowgar v. Commissioner Of Social Security Administration*; *Cowger v. Astrue*).^1^

## Bidirectional Scales and the Interpretation of Low Scores

Many human traits and symptoms are more or less normally distributed, and scores that deviate from the mean in either the high or low direction are equally “abnormal” in the statistical sense. Similar to measures of cognitive ability, where both high and low scores are meaningful, some measures of mood or personality also return a near normal distribution of scores, where high and low scores are of equal significance (McCrae et al. 2010; Russell et al. 2002). Scales which produce meaningful high and low scores are sometimes called “bipolar scales” (Widiger 2011), but to avoid confusion with the diagnosis of the same name, the term “bidirectional scales” will be preferred here. Recent research has suggested that conceptualizing psychological measures as being unidirectional may be mistaken, as maladaptive traits can be observed in patients with scores at both ends of a scale’s distribution (Pettersson et al. 2014). For example, one study concluded that on a neuroticism scale (measuring high negative affectivity), a low score was suggestive of the glib and fearless traits seen in psychopaths. Thus, both very high and very low scores on this scale were indicative of psychological dysfunction (Widiger 2011).

Bidirectional scales would appear to be of particular interest in the assessment of patients with chronic pain, or somatoform or somatic symptom disorders. This is because unusually low scores on some measures may suggest denial or suppressed report, and numerous studies have associated cognitive and emotional suppression with heightened psychophysiological reactivity and symptom report.

Studies have found that thought suppression increases the occurrence of obsessive ruminations about the very subject that the patient is trying to ignore (Wegner 1994; Wegner and Lane 2002). The avoidance of talking about strong feelings or important experiences has also been associated with autonomic arousal. For example, studies have found that suppressed anger increased blood pressure more than manifest anger (Vogele et al. 1997) and is also associated with increased pain (Quartana et al. 2010). The suppression of negative emotions is also associated with compromised functioning of the immune system (Petrie et al. 2002; Petrie et al. 1998), while emotional inhibition and reports of unusually low stress are associated with elevated levels of muscular bracing and myofascial pain (Traue 2002). Other studies have demonstrated that emotional repression predicts poor outcome following multidisciplinary treatment for chronic pain (Burns 2000), higher levels of cardiac reactivity (Burns et al. 1999), hypertension (Gleiberman 2007), and with behavioral signs of anxiety (Giese-Davis et al. 2014). A factor analytic study of patients with chronic pain identified one factor consisting of patients with high pain and disability but an absence of emotional distress (Burns et al. 2001). Patients who suppress emotions are also less likely to recall undesirable information about their health (Millar 2006). Overall, while the interpretation of low scores is the cornerstone of neuropsychological assessment, the interpretation of low scores/low reports is often overlooked when assessing patients with chronic pain.

Diagnostically, unusually low affective reports have been associated with a personality construct called “alexithymia,” meaning “without words for feelings” (Sifneos 1996). Research has established that alexithymia includes difficulty identifying or describing feelings, externally oriented thinking, and a limited capacity for imagination (Lumley et al. 2007). Two studies have concluded that there is a consistent link between alexithymia and somatization (Allen et al. 2011; Bailey and Henry 2007), while other studies concluded that alexithymia contributes to the emergence of somatic symptoms in major depression (Gulec et al. 2013), to somatization after brain injury (Wood et al. 2009), to unrecognized affective distress associated with pain (Lumley et al. 2002), to increased illness behavior (Lumley et al. 1997), and to stronger electrodermal response in biofeedback (Friedlander et al. 1997). This relationship between alexithymia and somatization was also supported by the findings of a large population study (Mattila et al. 2008). Although neither the DSM-5 (American Psychiatric Association 2013), DSM-IV (American Psychiatric Association 2000), nor the ICD-10 (World Health Organization 2010) mentions alexithymia, all note that some somatizing patients may exhibit unexpectedly low levels of affective distress, which is consistent with the above.

As an example of the effects of unrecognized affective distress, a patient who is having an anxiety attack could state emphatically that the symptoms are not due to severe stress or anxiety and instead assert that she/he is having a heart attack. In such a scenario, the patient may report extremely high physical distress but deny or be unaware of the emotional origins of the symptom. The research reviewed above suggests that the denial of the affective component may actually increase the somatic component of the symptoms, and this suggests a need for the assessment of low affective scores.

Although some of the BHI 2 scales exhibit a truncated distribution of scores below the mean, and a positive skew (e.g., Pain Complaints, Substance Abuse, and Survivor of Violence), some are close to being normally distributed, with other scales exhibiting a negative skew (e.g., Defensiveness, Anxiety, and Perseverance) (Table 1). One BHI 2 scale, Perseverance, is negatively skewed to the degree that negative T scores are possible (which occurs when a T score is more than 5 standard deviations below the mean). Statistically, scores which are 5 standard deviations below the mean are just as “abnormal” as scores which are 5 standard deviations above, and the studies reviewed above suggest that such unusually low scores might be as clinically meaningful as high ones. The interpretation of such low scores is sometimes less clear, though.

**Table 1:** BHI 2 scale reliability, skew and correlations with select measures

	Cronbach’s alpha	Test-retest stability	Bidirectional skew	Correlation with select MMPI-2 measures		Correlation with select MCMI-III measures		Correlation with other measures	
Self-disclosure	.97	.94	.061	F–K index	.69	Disclosure	.62		
Defensiveness	.83	.93	−.081	Profile elevation	−.62	Debasement	−.56		
Somatic Complaints	.93	.97	.848	Hy-ODANX	.76 .66 .74			McGill Pain Questionnaire	.74
Pain Complaints	.85	.95	.756					Scored Pain DrawingMcGill Pain Questionnaire	.70 .61
Functional Complaints	.82	.92	.335					SF-36 FunctionMBMD Pain SensitivityMBMD Psych Referral	−.64 .52 .52
Muscular Bracing	.84	.94	−.106	ANX	.65				
Depression	.91	.93	.512	D	.70	Dysthymic Major Depression	.71 .67		
Anxiety	.83	.90	−.108	ANX	.54				
Hostility	.89	.88	.622	ANG	.67				
Borderline	.86	.88	.476	Neg Tx Indicator	.66	Borderline	.62		
Symptom Dependency	.82	.88	−.034	Hy-OAPS	.54 .44				
Chronic Maladjustment	.77	.94	.238	Pd	.46	Antisocial Alcohol Dependence	.62 .57		
Substance Abuse	.75	.94	1.137	AAS	.55	Alcohol Dependence	.40		
Perseverance	.81	.93	−.167	Ego Strength Neg Tx Indicator	.51 −.62				
Family Dysfunction	.81	.92	.478	FAM Pd	.70 .58				
Survivor of Violence	.79	.96	.560	FAM	.55				
Doctor Dissatisfaction	.84	.88	.244						
Job Dissatisfaction	.88	.97	.190					Minnesota Satisfaction Questionnaire	−.64

Adapted from Bruns and Disorbio (2003) and Millon et al. (2010)

While moderately low scores on the BHI 2 or other psychological measures may reflect that the patient is coping unusually well, as scores become very low or extremely low, a different type of adjustment problem may be indicated. For example, an utter absence of any perceived affective distress could be explained as being attributable to extraordinary resilience, or alternately to psychopathy, alexithymia, denial, or dissociation. In the clinical setting, as each of these alternatives is associated with different behaviors, behavioral observations and the patient’s history might be needed to help determine which interpretation is correct.

On all of the BHI 2 scales, the lowest possible raw score is 0, and this can be a useful benchmark in interpretation. For example, in order for a patient to receive a raw score of 0 on Hostility, the patient must have strongly disagreed with 16 items having to do with anger. If, in an interview, a patient denied ever having any angry thoughts, feelings, or behaviors 16 times, it would seem remarkable. Consequently, scores this low on the BHI 2 are empirically unusual, and often intuitively and clinically meaningful. Even so, the interpretation of low psychological scales scores is often problematic.

A final caveat here is that as a rule, there is far less research on psychological measures about the interpretation of low scores as opposed to high scores.

## BHI 2 Interpretation Using Multiple Norm Groups

Any standard score compares the raw score of the individual to some reference group, and that comparison must be referenced when interpreting the score. When interpreting a patient’s score, there are two questions to answer:

The first question to answer is “how does this patient compare to other similar patients?” To the extent that a particular patient is atypical, normal treatment protocols may not apply. Further, knowledge of how a patient differs from a typical patient can inform treatment decisions and assist in the process of selecting patients for medical treatments. As a general psychometric principle, the closer the norm group to a patient’s status and circumstances, the more relevant the resulting score (American Educational Research Association, American Psychological Association, National Council on Measurement in Education, and Joint Committee on Standards for Educational and Psychological Testing (U.S.) 1999).

A second question that is sometimes overlooked is “how does this patient compare to the average healthy person?” To assess the severity of a patient’s psychological condition, or the degree to which a patient has been harmed by an injury, a comparison to a healthy state is required. This is because the effect of a medical condition cannot be seen in a comparison to other medical patients, as all have been affected by a similar loss of health. For example, if a patient who has had a recent traumatic amputation reports an average level of PTSD compared to other patients with traumatic amputations, this average level of PTSD does not mean that no PTSD is present. Instead, it means that compared to other amputees, the level of PTSD is similar. If this same patient’s PTSD score was compared to the average person in the community, however, it may be that the patient’s level of distress now appears quite elevated. Overall, it can be seen that if the norm group is extreme, a patient with an extreme problem will appear normal relative to that group, but that is not the same as a state of health. This leads to the somewhat counterintuitive interpretive dilemma if a PTSD scale utilized a norm group consisting of extremely traumatized subjects, an average score would be positive for PTSD.

A few tests used for the assessment of medical patients attempt to address the questions above by having multiple norm groups, notably the Minnesota Multiphasic Personality Inventory-2 Restructured Form (MMPI-2-RF), the Millon Behavioral Medicine Diagnostic (MBMD), and the BHI 2. However, these tests all address this matter in different ways, with different advantages and disadvantages. For forensic assessment, the MMPI-2-RF includes disability litigant norms and spinal surgery norms (Ben-Porath and Tellegen 2011), while the MBMD utilizes both general medical and chronic pain norms (Millon et al. 2010). The BHI 2's use of norm groups is somewhat different than these measures, however (Bruns and Disorbio 2003), and is described below.

As opposed to utilizing a highly specific norm group, the BHI 2 patient norm group utilizes a more diverse cross section of patients in treatment for pain or injury, consisting of an approximately equal number of patients with acute injuries and chronic pain. This group included spinal surgery patients, nonspinal surgery patients, interventional pain medicine patients, chronic pain patients, work hardening patients, acute pain patients, and brain injury patients, with 26 % being in litigation. These patients were obtained from 90 sites in 30 US states, recruiting patients from both facilities that treated chronic pain and that treated acute injuries. Patients were stratified to match US Census demographics (Bruns and Disorbio 2003). This approach has broad applicability, because patients referred for spinal presurgical evaluations might have acute conditions, while patients referred prior to spinal cord stimulation are invariably suffering from chronic pain, and litigation is common in the workers’ compensation and personal injury systems. Overall, this approach produced a psychometrically representative but less extreme normative sample that represented an attempt to depict the average patient in treatment for pain or injury. The second BHI 2 norm group is the community norm group, which was also stratified to match US Census demographics (Bruns and Disorbio 2003).

In addition to the two main normative groups, the BHI 2 also includes eight smaller, narrowly focused reference groups. These are chronic pain, head injury/headache, neck injury, upper extremity injury, low back injury, lower extremity injury, fake good, and fake bad. These additional more narrowly focused normative groups are integrated into the interpretive analysis and used to address specific questions if needed.

The BHI 2 differs from other multi-norm tests in that as opposed to having the user choose between alternative norm groups, the BHI 2 patient and community norm groups are integrated into a single continuum. This is accomplished in the following manner, using pain as an example: Not surprisingly, the average patient in treatment for pain or injury reports more pain than does the average nonpatient in the community. Even so, there are some Pain Complaints scores on the BHI 2 that fall within the normal range for both the patient and community norm groups. These levels of pain are designated as “average scores.” As scores on the Pain Complaints scale increase, at some point, the level of pain reported will be unusual for a healthy person but still commonly seen in patients in treatment for pain. As the level of Pain Complaints increases further, at some point, it will also become an unusual level of pain reports for a patient as well.

**Fig. 2:**
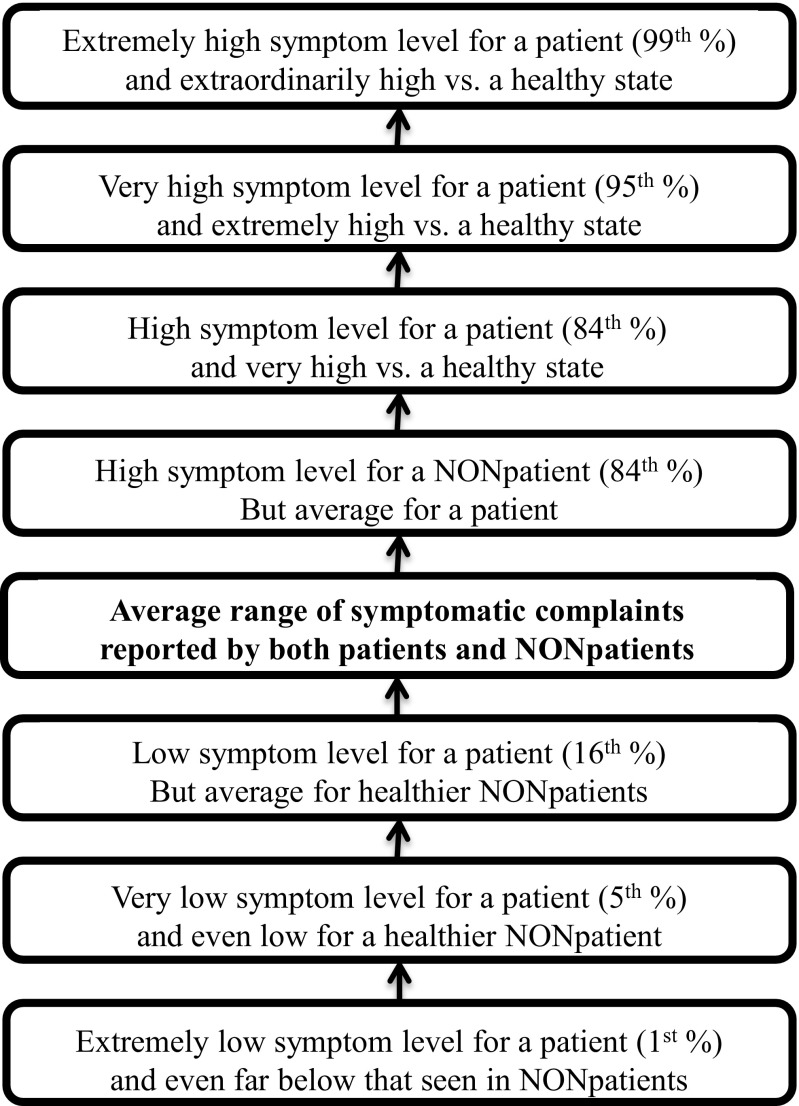
The interpretive continuum of BHI 2 scale scores using integrated patient and nonpatient norm groups

**Fig. 3:**
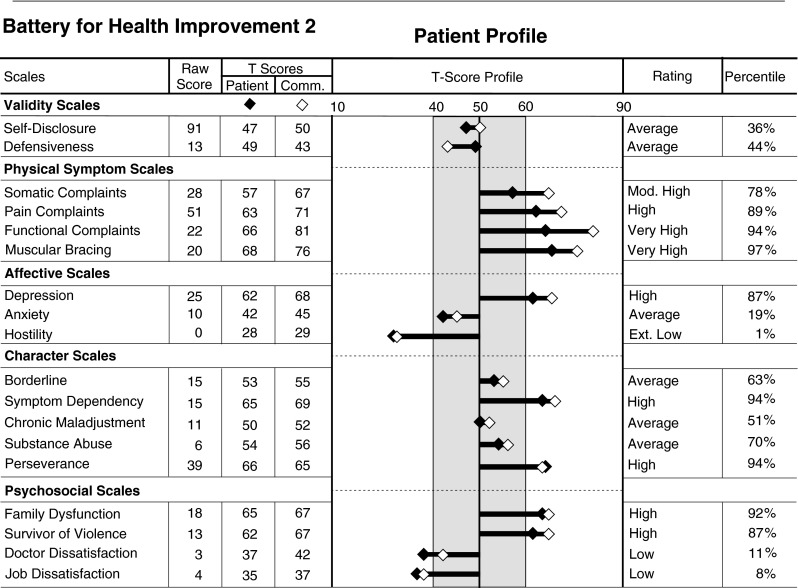
BHI 2 profile of patient with chronic pain

Conversely, with regard to interpreting low reports of pain, a patient may report less pain than does the average patient in treatment for pain or injury. As the Pain Complaints scores fall still lower, however, the level of pain will not only be below the typical patient in treatment, but also below that seen in the typical healthy person, which would be particularly unusual. Using this algorithm, the Pain Complaints scale integrates both community and patient norms into a single continuum (Fig. 2). The BHI 2 integrated norm profile is interpreted as follows, utilizing the profile shown in Fig. 3:

1. The BHI 2 profile uses a deviation bar chart, where the length of the bar represents how far the patient’s score deviates from the mean score. Thus, statistically, the bar is a visual representation of a *z* score. The longer the bar, the more the score deviates from the mean in either a positive or negative direction.
2. T scores within the shaded T = 40 to T = 60 range are typical for the normative patient and community samples, and approximately 68 % of the samples scored within this range.
3. Patient T scores are represented by black diamonds. A black diamond outside the average range indicates a level of symptoms that is unusual for patients with pain and injury. For unusual patients, the typical medical protocol may not apply.
4. Community T scores are represented by white diamonds. A white diamond outside the average range indicates that the patient is different than the average nonpatient in the community.
5. The Somatic Complaints scale score has the white diamond outside the average range (indicating a high score for community members), and the black dot inside the average range (indicating an average score for patients). Thus, while patients commonly report this level of Somatic Complaints, healthy people do not.
6. In this profile, both diamonds are outside the average range for the Pain Complaints scale. This indicates that the patient is not only reporting more pain than the average person in the community, but also reporting more pain than the average patient in treatment for pain or injury.
7. The Hostility scale score was the lowest score observed in this administration, being at the first percentile, indicating a remarkable absence of any reported anger or irritability. It should be noted that the raw score on this scale was 0, the lowest possible score. Thus, the patient must have strongly disagreed with all 16 items, which is statistically very unusual.
  In general, community norms are more sensitive with regard to detecting low levels of problematic symptoms than are the patient norms, but at the risk of increased false positive findings.

## BHI 2 Scale Interpretation

The following section provides an overview of the BHI 2 scales. This will include a brief synopsis of the scales’ rationale and development process, validity research, and interpretive considerations. Except as otherwise noted, the information presented below was derived from the BHI 2 validation study (Bruns and Disorbio 2003). An overview of each scale’s reliability, and its correlates and bidirectional skew is summarized in Table 1.

### Validity Scales and Methods

#### Validity Items

##### Development

The BHI 2 utilizes two separate validity conditions to determine whether or not a protocol can be meaningfully interpreted. The first condition has to do with bizarre responding, while the second has to do with failure to respond (i.e., leaving items blank). With regard to the first condition, the BHI 2 contains four Validity Items, which have extreme or bizarre content and are rarely endorsed. While a patient with a thought disorder might endorse one or more of these items, an alternate interpretation is that the patient was responding in the aberrant direction on these items due to illiteracy, random responding, visual/cognitive problems, or poor motivation.

##### Scale Interpretation

When a patient endorses one or more of the Validity Items, the item is printed out for the clinician to review along with a validity caution. If three or more of these items are endorsed, the entire BHI 2 is regarded as invalid due to bizarre responding. If this method determines that the BHI 2 administration is valid overall, the scales are next tested individually.

#### Blank Responses

##### Method

The second validity condition occurs when a patient fails to respond to a number of items. Based on this method, the BHI 2 scales are invalidated one at a time. If any scale has 25 % or more of its items left blank, that scale is judged to be invalid and is not scored. This allows the BHI 2 to invalidate one scale at a time based on the blank item condition, rather than invalidate the entire test if one scale was left blank. The BHI 2 computer algorithms will generate an interpretive report of the information available if one or more scales are valid. This reduces the risk that a test administration will produce no usable results. If all 18 scales are invalid due to blank items, then the entire BHI 2 protocol is invalid. Note that if the patient is not in the workforce now or at the time of onset of the condition, the test instructs the patient to leave the Job Dissatisfaction scale blank. This scale is then designated as invalid, while the remainder of the BHI 2 scales are interpreted.

#### Defensiveness

##### Development

The Defensiveness scale was developed empirically to assess both positive and negative response distortions using the following method. Patients in treatment for pain and injury were recruited and asked to subtly fake the BHI 2 good or bad, being cautioned that if their faking was too obvious, they might be caught and they should try to avoid that. This method was used as research has shown that if patients think that they will be undergoing assessment for faking, they are able to feign deficits in a way that is both less exaggerated and more believable (Youngjohn et al. 1999). Both groups of patients were then asked 475 questions, and items that were able to significantly differentiate both fake good and fake bad scenarios from patients were selected for the BHI 2 Defensiveness scale (Bruns and Disorbio 2003).

##### Scale Interpretation

High scores suggest a positive bias (faking good), and low scores a negative bias (faking bad). This scale is highly bidirectional and negatively skewed. Patients who are low in Defensiveness are describing their life and circumstances as terrible, while patients who are high in Defensiveness describe their life and circumstances as great. In either case, however, the Defensiveness scale does not contain items pertaining to personally sensitive information, so neither high nor low scores involve much self-disclosure. In the validity section of the BHI 2 report, unusual Defensiveness scores are compared to the two faking reference groups, producing a subtle fake good percentile rank or a subtle fake bad percentile rank. This makes it possible for the examiner to better interpret any response bias in the patient’s BHI 2 profile.

##### Validity Research

At cross-validation, the Defensiveness scale was able to significantly differentiate subtle fake good versus patient groups and subtle fake bad versus patient groups significantly at *p* < .001. The Defensiveness scale correlated to −.56 with the MCMI-III Debasement (Z) scale, and to −.62 with the MMPI-2 Profile Elevation. Profile Elevation in turn is strongly associated with the MMPI 2 Disability Profile (Gatchel et al. 2006). High and low scales here were able to identify those asked to subtly bias the information presented in positive and negative directions, respectively.

#### Self-Disclosure

##### Scale Development

The Self-Disclosure scale was developed to assess how much a patient was willing to disclose about internal psychological distress. The Self-Disclosure scale was developed from a broad index of BHI 2 items pertaining to psychological distress and dysfunction, which excluded items about physical symptoms or social conflicts. It is conceptually similar to the MCMI-III Disclosure scale.

##### Scale Interpretation

High scores indicate that the patient is disclosing an unusually high level of information about psychological dysfunction. In the clinical setting, patients with high Self-Disclosure scores are likely to be very open about conveying their internal psychological distress and dysfunction, sometimes to the point of self-debasement, and are often seeking help. Low scores suggest an unusual absence of any reported psychological difficulties. Patients with low levels of Self Disclosure are psychologically more guarded and private, may have concerns about who has access to the information that will be disclosed, and might not be motivated to share much personal information with the examiner.

##### Validity Research

At cross-validation, the Self-Disclosure scale was able to significantly differentiate fake good versus patient groups and fake bad versus patient groups significantly at *p* < .001. The Self-Disclosure scale is correlated to .66 and .62 with the MCMI-III Disclosure (X) and Debasement (Z) scales, respectively, and to .69 with the MMPI-2 Dissimulation (F–K) index, to .58 with the Infrequency (F), and to −.57 with the Correction (K) scale. Very high BHI 2 Self Disclosure scores (indicating a very high level of reported psychological distress) have been identified as a possible indication of malingering (Rogers 2008).

### Physical Symptom Scales

The BHI 2 is a biopsychosocial inventory that assesses physical, psychological, and social variables. On the BHI 2, the Pain Complaints, Somatic Complaints, Functional Complaints, and Muscular Bracing scales assess physical symptomatology. The BHI 2 test administration begins with the Pain Complaints scale, followed by the Somatic Complaints scale. Since the presenting concern of medical patients is their physical symptoms, this begins the test administration with the likely area of their primary concern. It was hoped that this would reduce patient resistance to completing the inventory.

#### Pain Complaints

##### Development

Pain is assessed in two primary ways in clinical settings: numerical rating scales and pain drawings. At the time of this writing, a Medline search for “VAS” or “NRS” and “pain” yielded 13,664 articles, demonstrating that this approach to pain assessment is ubiquitous. In contrast to uni-dimensional ratings, scored pain drawing methods rely entirely or in part on the number of body sites with pain (Takata and Hirotani 1995), and widespread pain predicts disability (Gerdle et al. 2008; Grotle et al. 2010; Kamaleri et al. 2009; Overland et al. 2012), inactivity (McBeth et al. 2010), complaints of nonmusculoskeletal medical problems (Kadam et al. 2005), greater medical utilization (Kadam et al. 2005), autonomic dysfunction (McBeth et al. 2007), occupational disability (Mayer et al. 2008), Waddell signs (Chan et al. 1993), treatment outcome (Takata and Hirotani 1995; Voorhies et al. 2007), performance on isokinetic measures of strength and function (Ohnmeiss et al. 2000), and continuing chronic pain 12 years in the future (Andersson 2004). As pain has been identified by two systemic reviews as an important predictor of poor outcome from spinal surgery (Celestin et al. 2009; den Boer et al. 2006), a principle goal of the BHI 2 was to develop a multidimensional assessment of pain. The BHI 2 utilizes a hybrid approach to assess both pain intensity like a VAS/NRS, and pain distribution like a pain drawing. To accomplish this, the BHI 2 Pain Complaints scale asks about pain intensity in ten different body areas, which provides a composite score.

##### Interpretation

High scores on the Pain Complaints scale indicate widespread pain in multiple body areas. This pattern of diffuse pain complaints can be observed in patients with diffuse rheumatoid arthritis, fibromyalgia, and chronic pain generally. This diffuse pattern of pain is difficult to explain in patients with a localized injury and suggests the possibility that central sensitization^2^ may be contributing to pain perception. Low scores on the Pain Complaints scale indicate that the patient is unusually pain free, possibly suggesting stoicism or reluctance to share information about pain.

##### Validity Research

The Pain Complaints scale correlated to .70 with a scored pain drawing, and to .61 with the McGill Pain Questionnaire. The number of body areas with pain on this scale was also determined by one study to be a significant predictor of a failure to make functional improvements following interdisciplinary treatment (Freedenfeld et al. 2002). Patients who are faking good tend to get lower scores on Pain Complaints (Disorbio et al. 2014). Patients with both cancer-related and noncancer-related breakthrough pain also scored higher on Pain Complaints (Portenoy et al. 2010).

#### Other BHI 2 Pain Measures

##### Highest Pain, Lowest Pain, and Pain Now

Similar to the Brief Pain Inventory (Cleeland 2009), the BHI 2 asks the patient to rate his/her highest, lowest, and current pain levels. BHI 2 Highest Pain levels have been positively associated with breakthrough pain (Portenoy et al. 2010), with wanting pain medication (Bruns et al. 2013), with medication noncompliance (Bruns et al. 2013), with smoking (Fishbain et al. 2013), with pain catastrophizing (Bruns et al. 2013), and with both delayed sleep onset and frequent awakenings (Bruns and Bruns 2011).

##### Pain Range

Pain Range assesses the variability of the patient’s pain complaints by comparing the difference between the patient’s highest and lowest pain reports in the last month. If a patient’s Pain Range score is empirically low (<2), the patient is saying that his/her pain has been remarkably invariant over the course of the last month (i.e., “my pain never changes”), which is empirically unusual but would be consistent with the theory that chronic pain is often not a sensory experience, and has been more closely associated with memory (Apkarian et al. 2009). If Pain Range is high (>6), it raises the question as to what circumstances produce the unusual pain variation. One study found that pain variability is associated with depression and more severe pain (Zakoscielna and Parmelee 2013). However, the BHI 2 Pain Range measure has not been empirically investigated itself.

##### Pain Tolerance Index

The Pain Tolerance Index (PTI) is a BHI 2 measure of pain intolerance. The BHI 2 PTI score correlated significantly with depression, anxiety, somatization, quality of life, disability, pain interference, and with physical difficulties with functioning (Bruns et al. 2005). PTI norms and reliability have been developed (Disorbio et al. 2013).

##### Pain Diagnostic Category

**Fig. 4:**
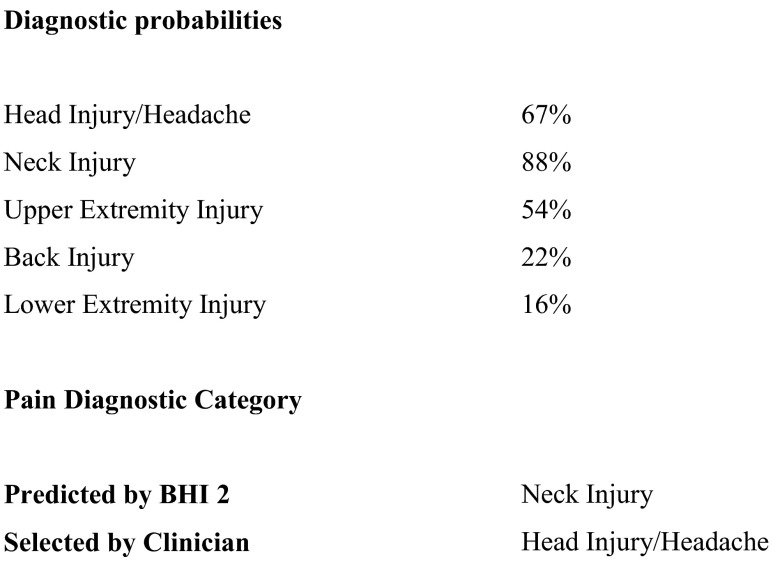
BHI 2 pain diagnostic analysis

In the clinical setting, pain drawings are judged by visual inspection with regard to the degree that they are displaying an “anatomical distribution” or not. In contrast, the BHI 2 assessment of the “anatomical distribution” of pain reports utilizes a computerized empirical approach. This analysis utilizes five BHI 2 pain normative groups, which are head injury/headache, neck injury, upper extremity injury, low back injury, and lower extremity injury. This approach mathematically compares the distribution of a patient’s 10 pain reports on the BHI 2 to patients in various diagnostic categories, using a series of discriminant functions. At cross-validation, this method accurately classified patients’ pain diagnostic category 81 % of the time (*p* < .0001) (Bruns and Disorbio 2003), and this can have some clinical utility. For example, Fig. 4 shows the Pain Diagnostic Category analysis for a patient referred for treatment for TBI-related headaches. However, the predicted pain diagnostic category was “neck injury,” as this distribution of pain reports was a better match for that diagnostic category. This could suggest alternative treatment approaches, or different causality of the pain symptoms.

#### Somatic Complaints

##### Scale Development

The items of the Somatic Complaints scale were selected so that they represented a cross section of the physical symptomatology associated with psychologically distressing conditions (e.g., somatization, physical symptoms of panic, generalized anxiety, and major depression). Research on the report of somatization symptoms shows that these symptoms are associated with chronic pain (Fishbain et al. 2009a) and disability (Harris et al. 2009). Another study found that somatization predicts suicidality even when depression is controlled for (Chioqueta and Stiles 2004). Somatization has also been identified by two systemic reviews as an important predictor of poor outcome from spinal surgery (Celestin et al. 2009; den Boer et al. 2006)

##### Scale Interpretation

A variety of medical and psychological conditions are associated with somatic distress. Depression, anxiety, and pain all have overlapping physical comorbidities in the form of physiological arousal, fatigue, difficulty concentrating, loss of libido, and similar symptoms. A high score on the Somatic Complaints scale suggests that the patient is very somatically distressed. This scale is only moderately bidirectional, but low scores nevertheless suggest that the patient is unusually symptom free and could be concerned about reporting physical weaknesses or vulnerabilities.

##### Validity Research

High BHI 2 Somatic Complaints scale scores are associated with having an uncertain diagnosis (Fishbain et al. 2010a), with fearing that the physician has missed something important (Fishbain et al. 2010a), with feeling entitled to medical care at no cost (Fishbain et al. 2014c), with frequent suicidal thoughts (Fishbain et al. 2012b), with having a suicide plan (Fishbain et al. 2012b), with a history of suicide attempt (Fishbain et al. 2012b), with litigious ideation in patients with acute pain (Fishbain et al. 2008), and with pain severity (Fishbain et al. 2014d). Recent research has hypothesized that somatic complaints such as these are perhaps better conceptualized as comorbidities of chronic pain, which are closely associated with the pain syndrome (Fishbain et al. 2014a).

#### Functional Complaints

##### Scale Development

In contrast to measures such as the Oswestry (Fairbank and Pynsent 2000), or the SF-36 Function Scale (Ware et al. 1993), which measure physical impairment (such as how much you can lift or how far can you can walk), the BHI 2 Functional Complaints scale is focused on assessing the perception of disability. This involves the belief that one is unable to be gainfully employed and unable to adequately perform activities of daily living, whatever that might entail. Functioning was identified by two systemic reviews as an important predictor of poor outcome from spinal surgery (Celestin et al. 2009; den Boer et al. 2006) and is the outcome goal for most treatments in physical medicine.

##### Interpretation

Patients with high scores on Functional Complaints perceive themselves as being disabled and unable to function at work, home, or both. Functional Complaints is a bidirectional scale, and patients with low scores are denying that they have functional limits. When possible, the interpretation of the Functional Complaints scale should be performed in conjunction with whatever medical information is available about the patient’s objective ability to function. If there are large discrepancies between the patient’s perceived and actual physical abilities, the greater these discrepancies, the more likely it is that the perceptions of disability are attributable to an unrealistic cognitive appraisal of physical limitations.

##### Validity Research

High scores on the BHI 2 Functional Complaints scale are associated with a perceived need of pain medication (Bruns et al. 2013), feeling entitled to medical care at no cost (Fishbain et al. 2014c), and breakthrough pain (Portenoy et al. 2010). The BHI 2 Functional Complaints scale has strong negative correlation (−.62) with the SF 36 Function scale, which measures functional ability rather than disability (Bruns and Disorbio 2003).

#### Muscular Bracing

##### Scale Development

Muscle tension has been called the most discriminative symptom of generalized anxiety disorder. However, muscle tension has a complex relationship with anxiety, and perceptions of muscle tension may be either objectively true or subjectively perceived (Pluess et al. 2009). Perceived muscle tension is associated with diffuse subjective feelings of “tension,” which may be physical or affective in nature. The Muscular Bracing scale was designed to assess this experience of feeling tense. The items on the scale were generated based on item content, refined using an internal consistency method, and subsequently investigated in the empirical studies listed below.

##### Interpretation

Patients with high scores on muscular bracing are reporting perceptions of chronic problems with muscle tension and being perceived by others as tense persons. This may be objectively true, or may correspond with a subjective sense of tension, which is perceived as being muscular in nature but may actually be more closely associated with anxious arousal. The Muscular Bracing scale is almost perfectly normally distributed, with low scores indicating reports of a complete lack of muscle tension and being perceived by others as being unusually relaxed.

##### Validity Research

The Muscular Bracing scale correlated to .65 with the Anxiety (ANX) scale of the MMPI-2. In addition, the Muscular Bracing scale correlated to .68, .59, .60, and .52 with the BHI 2 Somatic Complaints, Anxiety, Depression, and Hostility scales, respectively. High BHI 2 Muscular Bracing scale scores are associated with having an uncertain diagnosis (Fishbain et al. 2010a), with pain catastrophizing (Bruns et al. 2013), with a history of childhood molestation in females with chronic pain (Fishbain et al. 2014b), and with wanting to die because of pain (Fishbain et al. 2012a). In one study, Muscular Bracing was the strongest predictor of both delayed sleep onset and of unrefreshing sleep (Bruns and Bruns 2011).

### Affective Scales

It has been estimated that about 30 % of the variance in psychological tests results was attributable to difficulties with physical functioning (Naliboff et al. 1982), and as a result, many psychological tests are influenced heavily by a disease or injury, its sequelae, and medication side effects as opposed to the patient’s psychological condition (Turk and Melzack 1992). When constructing the BHI, controlling for such false positive findings was a priority.

The assessment of depression and anxiety is particularly confounded by medical symptomatology. In order to control for this, the BHI 2 includes two separate measures for both depression and anxiety. The BHI 2 Depression and Anxiety scales focus on thoughts and feelings, and measure what could be thought of as phenomenological depression/anxiety. Two separate measures, Vegetative Depression and Autonomic Anxiety, are subscales of Somatic Complaints and include the physical symptoms of depression and anxiety, respectively (e.g., fatigue, weight change, loss of libido, and racing heart). The advantage of this strategy is to reduce the risk of false positive findings on the Depression and Anxiety scales due to physical symptoms of medical illness. The disadvantage of this approach is that by dividing both depression and anxiety assessment into two separate scales, neither scale contains the entire diagnostic criteria. However, when both Depression/Vegetative Depression measures are elevated, or Anxiety/Autonomic Anxiety measures are elevated, this suggests that cognitive, affective, and physiological symptoms of these conditions are all present.

### Depression

#### Scale Development

Depression is an important variable to measure in patients with chronic pain, as there is a high prevalence of depressive symptoms in those with pain. The BHI 2 Depression scale differs from most others in that it also includes items pertaining to sad thoughts and feelings associated with physical health problems. Depression was identified by two systemic reviews as an important predictor of poor outcome from spinal surgery (Celestin et al. 2009; den Boer et al. 2006).

#### Scale Interpretation

A high score on the BHI 2 Depression scale indicates that the patient is aware of feeling subjectively sad and depressed and is reporting feelings of helplessness, difficulties with adjusting to health changes, bitter disappointment with health, anhedonia, and thoughts of suicide. In contrast, this scale is moderately bidirectional, and a low score on Depression indicates that the patient is denying having sad feelings or negative thoughts and is reporting believing that the future seems bright, feels energetic and optimistic, and feels that life is easy and satisfying. If a patient was reporting a serious medical problem, low scores would seem counterintuitive and could possibly reflect denial or alexithymic tendencies.

#### Validity Research

The BHI 2 Depression scale correlated to .70 with the MMPI-2 Depression (D) scale, to .71 with the MCMI-III Dysthymic Disorder (D) scale, and to .67 with the MCMI-III Major Depression (CC) scale. High BHI 2 Depression scores have been associated with wanting to die (Fishbain et al. 2012b), with wanting to die because of pain (Fishbain et al. 2012b), with wanting to die because life is hard (Fishbain et al. 2012b), with frequent suicidal thoughts (Fishbain et al. 2012b), with having a suicide plan (Fishbain et al. 2012b), with thoughts of suing a physician in acute patients (Fishbain et al. 2008), with thoughts of killing a physician (Bruns et al. 2010), with feeling entitled to not have to wait to see a physician (Fishbain et al. 2014c), with failure to make functional improvements during interdisciplinary treatment (Freedenfeld et al. 2002), and with breakthrough pain (Portenoy et al. 2010).

#### Anxiety

##### Scale Development

The construct that the BHI 2 Anxiety scale might be most closely associated with is DSM-5 Illness Anxiety, and to a lesser extent with fear avoidance. This scale includes items pertaining to worried and fearful thoughts and feelings associated with physical health problems, illness, and injury, including fears of dying. Anxiety was identified by two systemic reviews as an important predictor of poor outcome from spinal surgery (Celestin et al. 2009; den Boer et al. 2006)

##### Interpretation

High scores on the Anxiety scale suggest a person who is feeling fearful, restless, engages in self-protective behaviors, can have hypochondriacal fears of illness, is prone to worrying, has multiple health fears, and worries that the medical condition may somehow lead to death. Patients with high scores also have social fears as well. In contrast, patients with low scores on the anxiety scale are reporting being untroubled by worries or health concerns, not worrying about behaviorally guarding themselves so as to avoid injury, and not worrying about contracting illnesses. The BHI 2 Anxiety scale has a moderately bidirectional distribution.

##### Validity Research

The BHI 2 Anxiety scale correlated to .54 with the MMPI-2 Anxiety (ANX) scale. High BHI 2 Anxiety scale scores are associated with having an uncertain diagnosis (Fishbain et al. 2010a), with fearing that the physician has missed something important (Fishbain et al. 2010a), and with feeling entitled to not have to wait to see a physician (Fishbain et al. 2014c).

#### Hostility

##### Scale Development

The assessment of hostility was believed to be an important scale to include on the BHI 2, as anger and anxiety are the two components of the *fight or flight* response. A review of the concept of hostility revealed that it is broken down into three components: angry feelings, cynical thoughts, and aggressive behaviors (Barefoot 1992). The BHI 2 Hostility scale was constructed with items representing all three of these components.

##### Interpretation

High scores on the Hostility scale suggest a patient who has cynical thoughts about others, is prone to anger and irritability, is quick to take offense, and can respond to frustration with an irritable or even belligerent manner. Low scores on the Hostility scale indicates that the person is reporting having faith in the kindness of others and being patient, long-suffering, and easy-going or passive. As with low scores generally, unusually low reports of hostility can be the product of an accurate self-report, denial, dissociation, or alexithymic tendencies.

##### Validity Research

High BHI 2 Hostility scores have been associated with feelings of anger (Fishbain et al. 2011c), with chronic anger (Fishbain et al. 2011c), with thoughts of killing a physician in patients (Bruns et al. 2010), with violent ideation generally (Bruns and Disorbio 2000; Bruns et al. 2007), with thoughts of homicide/suicide (Fishbain et al. 2011b), with frequent suicidal thoughts (Fishbain et al. 2012b), with thoughts of suing a physician (Fishbain et al. 2007), with feeling entitled to not have to wait to see a physician (Fishbain et al. 2014c), with wanting to be totally pain free (Bruns et al. 2013), and with being oppositional (Bruns et al. 2013).

### Characterological Dysfunction Scales

The prevalence rate of personality disorder in the general population has been estimated as being between 5.9 and 13.5 % (Dersh et al. 2002). In contrast, in five studies of patients with chronic pain, the prevalence rate of personality disorder ranged from 40 to 77 % (Dersh et al. 2006; Fishbain et al. 1986; Large 1986; Okasha et al. 1999; Polatin et al. 1993). The DSM-5 uses the concept of the “general personality disorder” to broadly describe the maladaptive traits that underlie all personality disorders (American Psychiatric Association 2013). The criteria for the general personality disorder involve two clusters of traits, with the first cluster of traits being those involving emotional dysfunction and interpersonal conflict (represented by the BHI 2 Borderline scale), and the second cluster of traits involving a history of poor impulse control and impairment in social functioning, occupational achievement, and other areas of function (represented by the BHI 2 Chronic Maladjustment scale).

With regard to the assessment of personality disorders in patients with chronic pain, a caveat here is that a recent study suggested that following the onset of chronic pain patients may exhibit increased characterological dysfunction, which may in turn decrease if the pain is treated effectively (Fishbain et al. 2006).

#### Borderline

##### Scale Development

By averaging the prevalence rates reported by eight separate studies, one study estimated that 30 % of patients with chronic pain also suffer from borderline personality disorder (Sansone and Sansone 2012). This study went on to conclude that patients with borderline personality report higher levels of pain, theorizing that these patients may have difficulty with self-regulating pain and may use the pain syndrome to solicit care from others. The items on the BHI 2 Borderline scale were generated based on item content. The Borderline scale items focus on aspects of this characterological disturbance that were thought to be especially relevant to the physical rehabilitation setting. This included the loss of identity, self-destructive behaviors, and a tendency to develop highly conflicted relationships with others (splitting). This scale was refined using an internal consistency method and subsequently investigated in the empirical studies listed below.

##### Interpretation

High scores on the Borderline scale indicate patients who report low self-esteem, difficulty regulating their moods, an intolerance for frustration, a history of conflicted relationships with others, and a tendency to punish themselves for their own perceived weaknesses or defects. This is a moderately bidirectional scale, and low scores reflect claims of an unusual absence of conflict or mood variations, which could be explained by excellent mental health and a strong support system, or alternately by an avoidance or denial of conflict.

##### Validity Research

The BHI 2 Borderline scale correlated to .62 with the MCMI-III Borderline scale, to .60 with the MMPI 2 Anxiety (ANX) scales, and to .61 with the MCMI-III Anger (ANG) and Family Problems (FAM) scales. High BHI 2 Borderline scores have been associated with wanting to die (Fishbain et al. 2012b), with wanting to die because life is hard (Fishbain et al. 2012b), with frequent suicidal thoughts (Fishbain et al. 2012b), with having a suicide plan Fishbain et al. (2012b), with a history of suicide attempt (Fishbain et al. 2012b), with a preference for death over disability (Fishbain et al. 2012a), with chronic anger (Fishbain et al. 2011c), with thoughts of killing a physician (Bruns et al. 2010), with violent thoughts generally (Bruns and Disorbio 2000), with thoughts of homicide/suicide (Fishbain et al. 2011b), with thoughts of suing a physician (Fishbain et al. 2007), and with medication nonadherence (Fishbain et al. 2010b).

#### Chronic Maladjustment

##### Scale Development

The BHI 2 Chronic Maladjustment scale utilizes items inquiring about a history of difficulties in school, unstable relationships, vocational instability, unstable living arrangements, financial irresponsibility, impulsivity, failed life plans, reckless disregard for safety, and incarceration. This scale has considerable diagnostic overlap with antisocial personality. The items of the Chronic Maladjustment scale do not tap the violent aspects of antisocial personality but do focus on the inability to attain common milestones of successful adult functioning seen in general, antisocial, and other personality disorders as well.

##### Interpretation

Patients with high scores on the Chronic Maladjustment scale have reported a history of irresponsible and impulsive behavior, and of failing to succeed in legal, financial, educational, employment, relationship, and other aspects of responsible adult life. These patients may be at greater risk for failing to succeed in a demanding rehabilitation program as well. This scale is moderately bidirectional, and low scores indicate that the patient is claiming success in achieving most common milestones of a stable adult life, and to be a responsible social achiever who lives a conventional life.

##### Validity Research

The Chronic Maladjustment scale was correlated to .62 with the MCMI-III Antisocial (6A) scale, to .46 with the MMPI-2 Psychopathic Deviate (Pd) scale, and to .57 and .55 with the MCMI-III Alcohol Dependence and Borderline scales, respectively. The Chronic Maladjustment scale was also associated with a preference for death over disability (Fishbain et al. 2012a), and noncompliance with medication (Bruns et al. 2013).

#### Symptom Dependency

##### Scale Development

The DSM-IV, DSM-5, and ICD 10 all discuss somatizing disorders with regard to the adoption of a dependent role, which revolves around the patient’s somatic complaints. With regard to somatic symptom disorders, the DSM-5 states that “health concerns may assume a central role in the individual’s life, becoming a feature of his or her identity and dominating interpersonal relationships” (American Psychiatric Association 2013; p. 311). In a discussion of somatoform disorders, the DSM-IV states this somewhat more succinctly, stating that some somatoform disorders can lead to “dependency and the adoption of a sick role” (American Psychiatric Association 2000; p. 495). The BHI 2 Symptom Dependency scale was not intended to assess dependent personality per se, but rather to assess how some patients utilize their symptoms to form dependent attachments. The items on the BHI 2 Symptom Dependency scale were generated based on item content and were refined using an internal consistency method and subsequently investigated in the empirical studies listed below. Passive coping was identified by two systemic reviews as an important predictor of poor outcome from spinal surgery (Celestin et al. 2009; den Boer et al. 2006).

##### Interpretation

Patients with high scores on the Symptom Dependency scale are reporting symptoms that are refractory to medical care, associated with life stressors, and for which they feel entitled to the support of others. This scale is moderately bidirectional, and low scores indicate reports that when suffering from medical problems, the patient wants to be left alone.

##### Validity Research

The BHI 2 Symptom Dependency scale is correlated to .54, .48, .44, and .44 with the MMPI-2 Hysteria–Obvious (HyO) scale, Anxiety (A) scale, Addiction Potential Scale (APS), and Negative Treatment Indicators (TRT) scales, respectively. High scores on the BHI 2 Symptom Dependency scale are associated with decreased likelihood of employment in the 6 months following interdisciplinary treatment (Freedenfeld et al. 2002), with medical entitlement (Fishbain et al. 2014c), with violent ideation (Bruns et al. 2007), with being demanding (Bruns et al. 2013), and with sleep disturbance and frequent awakenings (Bruns and Bruns 2011). Very high Symptom Dependency scores have been identified as a possible indication of malingering (Rogers 2008).

#### Substance Abuse

##### Scale Development

The majority of studies of risk factors for poor medical recovery list substance abuse as a concern (Bruns and Disorbio 2009), with up to half of patients hospitalized for traumatic injury being intoxicated at the time of the injury, and two thirds having a history of substance abuse (Corrigan 1995). The Substance Abuse scale is composed of two blocks of items. The first block inquires into a history of abusing alcohol and other substances, while a second set of items assesses current dependence on prescription medication. This latter block of items differentiates this scale from most other substance abuse scales. A remote history of substance abuse increases the risk that an injury will result in disability (Upmark et al. 1999).

##### Interpretation

A high score on the Substance Abuse scale indicates that the patient is admitting a *history* of difficulties associated with chemical dependency, and *current* problems with prescription medication. This admission does not mean that the patient is currently suffering from addiction, but it may increase the risk that the patient would revert to chemical dependency as a means of coping with a medical problem. This scale is marginally bidirectional, and a low score indicates that the patient denies that his or her use of substances has ever been inappropriate or caused problems.

##### Validity Research

The BHI 2 Substance Abuse scale correlated to .55 with the MMPI-2 Addiction Admission Scale (AAS), and to .40 with the MCMI-III Alcohol Dependence (B) scale. The content of the BHI 2 Substance Abuse scale differs from that of the aforementioned alcohol and drug addiction scales in that it does not have items about addictive personality traits and includes some items about addiction to prescription medication. High scores on this scale are associated with medication nonadherence (Fishbain et al. 2010b), and violent ideation (Bruns et al. 2007). A high score on this scale also predicted a decreased likelihood of employment in the 6 months following interdisciplinary treatment (Freedenfeld et al. 2002).

#### Perseverance

##### Scale Development

The concept behind the BHI 2 Perseverance scale combines the literature of several positive psychological variables, which are optimism (Novy et al. 1998), psychological hardiness (Callahan 2000; Kobasa et al. 1982), and self-efficacy (Bandura 1992). This scale contains items representing optimism, hardiness, and self-efficacy, was refined using an internal consistency method, and subsequently investigated in the empirical studies listed below

##### Interpretation

High scores on the Perseverance scale indicate that the patient is reporting self-discipline, emotional resilience, and proactive conduct. Very high scores are empirically unusual and may involve exaggerated virtue or stubbornness. Perseverance is highly bidirectional, with negative T scores being possible (which occurs when a T score is more than 5 standard deviations below the mean). Low Perseverance scores indicate that the patient is reporting poor self-discipline, poor emotional coping, dysfunctional conduct, and helplessness

##### Validity Research

The BHI 2 Perseverance scale correlates strongly (.51) with the MMPI-2 Ego Strength (Es) scale. These scales are similar in that the Ego Strength scale assesses traits that include being reliable, determined, and self-confident, while the Perseverance scale measures feelings of optimism, hardiness, and self-efficacy. The Perseverance scale is also negatively correlated (−.62) with the MMPI-2 Negative Treatment Indicators (TRT) scale. Extremely high Perseverance scores (thus claiming excessive virtue) have been identified as a possible indication of malingering (Rogers 2008). Low BHI 2 Perseverance scale scores are associated with feelings of anger (Fishbain et al. 2011c), with wanting to die because life is hard (Fishbain et al. 2012b), with frequent suicidal thoughts (Fishbain et al. 2012b), with a preference for death over disability (Fishbain et al.  2012a), with having an uncertain diagnosis (Fishbain et al. 2010a), with medication nonadherence (Fishbain et al. 2010b), and with fearing that the physician has missed something important (Fishbain et al. 2010a).

### Social Dysfunction Scales

The third part of the BHI 2 biopsychosocial assessment involves the assessment of social factors. The importance of social support for the medical patient was established by a meta-analysis, which found that a supportive family substantially improved patient adherence with treatment (DiMatteo 2004). Similarly, on the other hand, another study found that social support was associated with decreased patient stress and improved quality of life following surgery (Laxton and Perrin 2003). Conversely, other studies have found that somatization is more likely to occur when social conflicts are present (Liu et al. 2011). In the BHI 2 social dysfunction scales, we explored the primary social domains relevant to the injured patient, which are the relationships with the family, the physician, and the employer. Additionally, this part of the test assesses signs of a traumatic social history.

#### Family Dysfunction

##### Scale Development

The Family Dysfunction scale assesses the patient’s relationship to family, and the degree of support that may be available. When a patient is recovering from an injury or illness, typically it is the family to which the patient turns for support during this difficult time. However, if the family is cold, uncaring, or abusive, or if family relationships are highly conflicted, this increases the stress on the patient. Research suggests that a supportive family can facilitate recovery during a time when patients may be considerably less functional, and more reliant on others (Abbasi et al. 2012; Elkayam et al. 1996). In contrast, a dysfunctional or nonsupportive family (which is being reported when there is a high score on the Family Dysfunction scale) can make the patient’s circumstances during rehabilitation especially difficult, and might increase the risk of poor recovery.

##### Interpretation

High scores on the Family Dysfunction scale are indicative of patients who report feeling unloved, unsupported, mistreated, or angered by their families. Perceptions such as these may give rise to feelings of insecurity, isolation, and vulnerability in the injured or physically ill patient. Given the intensity of conflicts present, these patients may rely heavily on their medical caregivers for meeting their security and support needs.

##### Validity Research

The Family Dysfunction scale correlated highly (.70) with the MMPI-2 Family Problems (FAM) scale. High BHI 2 Family Dysfunction scale scores are associated with medication nonadherence (Fishbain et al. 2010b), and with a preference for death over disability (Fishbain et al. 2012a).

#### Survivor of Violence

##### Scale Development

A series of US Centers for Disease Control studies have demonstrated a relationship between adverse childhood experiences (ACE), and morbidity and mortality decades later (Felitti et al. 1998). Other studies have identified a relationship between ACE and the appearance in adulthood of back pain (Schofferman et al. 1993), poor surgical outcome (Schofferman et al. 1992), and conversion disorder or somatization (Andreski et al. 1998; Kaplan et al. 2013; Ozcetin et al. 2009). PTSD in adulthood has also been shown to be associated with chronic pain (Morasco et al. 2013). The Survivor of Violence scale is composed of items describing a variety of traumatic experiences occurring in both childhood and adulthood.

##### Interpretation

High scores on the Survivor of Violence scale indicate an extensive history of physically or psychologically traumatic experiences. Patients with high scores are reporting that they have survived multiple traumatic events in their past. This scale is only marginally bidirectional, and low scores indicate that the patient is not reporting any lifetime history of psychological trauma.

##### Validity Research

Despite the fact that the majority of the BHI 2 Survivor of Violence scale items make no reference to family, it nevertheless correlated to .72 with the BHI 2 Family Dysfunction scale, to .55 with the MMPI-2 Family Problems (FAM) scale, and to .54 with the BHI 2 Borderline scale. High scores on the Survivor of Violence scale were associated with a history of a suicide attempt (Fishbain et al. 2011), noncompliance with medication (Bruns et al. 2013), and with litigious ideation (Bruns et al. 2013).

#### Doctor Dissatisfaction

##### Scale Development

When developing the BHI 2, it was hypothesized that if the patient has a highly positive view of the physician, or even thinks the physician is OK, it is unlikely to impair prognosis. However, if a patient comes to hate the physician, and to see the physician as an unempathic agent of an uncaring health care system, who is incompetent and only in for the money, then that may threaten the recovery process.

##### Interpretation

High scores on the Doctor Dissatisfaction scale indicate an unusual level of frustration and anger with the medical profession. Patients with elevated scores are reporting perceptions of physicians as unempathic and incompetent. This scale is moderately bidirectional, and low scores indicate a positive description of the physician. While this could suggest a positive therapeutic alliance, it could also be attributable to a reluctance to criticize an authority figure upon which one is dependent.

##### Validity Research

High BHI 2 Doctor Dissatisfaction scale scores are associated with having an uncertain diagnosis (Fishbain et al. 2010a), with fearing that the physician has missed something important (Fishbain et al. 2010a), with medical entitlement (Fishbain et al. 2014c), with more high-risk patient behaviors than any other tested risk factor (Bruns et al. 2013), with thoughts of suing a physician (Fishbain et al. 2007, 2008), with thoughts of killing a physician (Bruns et al. 2010), and with thoughts of homicide/suicide (Fishbain et al. 2011b).

#### Job Dissatisfaction

##### Scale Development

Although the BHI 2 Job Dissatisfaction scale was created in the context of workers’ compensation and other disability systems, it also applies to patients who are in the workforce. If a patient is injured while at work, having pain can allow a worker to escape from an undesirable job, receive pay while off of work, resume work with reduced duties, force the employer to make accommodations, receive opioid pain medication, receive desirable treatments, and if they fail to recover, receive monetary compensation for not getting better. Thus, when recovery means return to work, Job Dissatisfaction can be a significant predictor of poor outcome. Job Dissatisfaction was identified by a systemic review as an important predictor of poor outcome from spinal surgery (den Boer et al. 2006).

One study found that job dissatisfaction was the strongest predictor of reporting a workplace injury (Bigos et al. 1992), and of subsequent higher levels of pain (Davis and Heaney 2000). The Job Dissatisfaction scale has four subscales, describing dissatisfaction with the company, supervision, coworkers, and with the intrinsic nature of the job itself.

##### Interpretation

Patients with high scores on the Job Dissatisfaction scale reported feelings of resentment toward their employer. These patients may have higher demands for accommodation and may be at risk for conflict with supervisors. If recovery means returning to an unpleasant or disliked workplace, it may interfere with the patient’s motivation in rehabilitation. This scale is moderately bidirectional, and low scores indicate that the patient is reporting feeling satisfied with his/her employment.

##### Validity Research

The BHI 2 Job Dissatisfaction scale is negatively correlated (−.64) with the Minnesota Satisfaction Questionnaire (MSQ). Although the MSQ does not tap the depth of animosity that the BHI 2 Job Dissatisfaction scale does, the two scales both measure job-related attitudes and feelings. A high score on this scale was associated with an increased likelihood of undergoing surgery in the 6 months following interdisciplinary treatment (Freedenfeld et al. 2002).

## The Brief Battery for Health Improvement 2

The BHI 2 has a companion short form, called the BBHI 2. While the longer BHI 2 takes 30 to 35 min to administer, the BBHI 2 can be administered in about 10 min. The BBHI 2 is composed of six scales: Defensiveness, Pain Complaints, Somatic Complaints, Functional Complaints, Depression, and Anxiety. Of these, three (Defensiveness, Pain Complaints, and Functional Complaints) are identical to the scales on the longer BHI 2. The other three scales (Somatic Complaints, Depression, and Anxiety) are shortened versions of the longer BHI 2 scales, which intercorrelated very highly with the longer versions. The BBHI 2 Somatic Complaints scale correlates with the BHI 2 version to .96, the BBHI 2 Depression scale correlates with the BHI 2 version to .95, and the BBHI 2 Anxiety scale correlates with the BHI 2 version to .91.

The BBHI 2 Somatic Complaints, Depression, and Anxiety scales are different from their longer BHI 2 counterparts in one important respect. In contrast to the longer scales on the BHI 2, which includes both transient “state” type items with more enduring “trait” type items, the shortened BBHI 2 scales focus on state items, as opposed to trait items. The rationale for this is that the BBHI 2 was intended to be useful in serial assessment, in order to track changes over the course of treatment. In order to do so, however, it was necessary to focus on state items (e.g., “I feel sad”) as opposed to trait items (e.g., “I attempted suicide in the past”). This is because state items are changeable, whereas trait items are not. By this focusing on state items, the BBHI 2 scales focused on changeable symptomatology, making them more sensitive to change in response to treatment. It should be noted that the BBHI 2 scales can be scored from the longer BHI 2 administration.

The BBHI 2 also screens for a wide variety of difficulties through the use of critical items. The psychological concerns screened for by the BBHI 2 include suicidality, addiction, psychosis, death fears, panic, dissociation, satisfaction with care, family problems, insomnia, home life problems, dysfunctional pain cognitions, compensation focus, and other concerns.

## Presurgical and Pre-opioid Assessments

In the forensic setting, examiners are sometimes asked to form an opinion about the prognosis of a patient for benefitting from a surgery or other treatment. To this end, the BHI 2 can be employed to produce scores for several different presurgical or pre-opioid treatment assessment protocols. These protocols were developed for various purposes by different methods, and each has their own unique strengths and weaknesses.

### Presurgical Psychological Assessments

From the standpoint of the strongest scientific evidence, the two presurgical protocols with the highest level of evidence are based on the systematic reviews of psychosocial risk factors conducted by den Boer and colleagues (den Boer et al. 2006), and by Celestin and colleagues (Celestin et al. 2009). These two studies produced very similar results and share two weaknesses. First of all, neither den Boer nor Celestin suggests any means of performing a presurgical assessment. Thus, while they are very informative theoretically, neither study made any suggestions about translating their findings into clinical practice.

A second weakness of the den Boer and Celestin studies can be understood by considering the conclusions of expert panels that made recommendations about presurgical psychosocial risk factors for spinal cord stimulators (Bruns and Disorbio 2009). This study reported a clinical consensus that the most important risk factors to identify were those having to do with severe psychopathology, such as being suicidal, homicidal, psychotic, and addicted. Unfortunately, there are no randomized controlled trials (RCTs) assessing the outcome of back surgery on patients who are also suffering from any kind of severe psychopathology. As there are no such studies in the literature, the den Boer and Celestin studies had nothing to review, and they made no recommendations about severe psychopathology. Because of these limits to the research literature, if a patient was diagnosed with antisocial personality, cocaine addiction, paranoia, homicidality, had threatened the physician, and had a history of making fraudulent medical claims to obtain opioids, that patient would have no psychosocial risk factors using either the den Boer or Celestin protocols.

In contrast to the den Boer and Celestin scientific studies, Block and colleagues (Block et al. 2003; Block et al. 2001; Epker and Block 2001) and Bruns and Disorbio (Bruns and Disorbio 2009, 2013) developed clinically oriented protocols. These protocols for the most part subsume the den Boer and Celestin criteria, while adding more serious signs of psychopathology. In contrast to den Boer’s and Celestin’s approaches, Block’s translational science method integrates psychological testing and educational information for psychologists about the surgical procedures involved, along with an integrated clinical algorithm.

Using a different methodology than Block and colleagues, the Bruns and Disorbio review produced a two-tiered set of risk factors coupled with a clinical assessment method. The first tier was of moderate risk factors similar to Block’s criteria in many respects, and like Block, it subsumed the den Boer and Celestin criteria. However, the Bruns and Disorbio study also identified a separate set of severe “exclusionary” or “primary” risk factors, any one of which is widely held to be so behaviorally disruptive as to create grave concerns about a patient’s ability to benefit from medical treatments (Bruns and Disorbio 2009). While there are no meta-analyses, systematic reviews, or RCTs of any of these primary risk factors, some empirical studies of these extreme risk factors have recently been published. These include studies pertaining to suicidal ideation (Fishbain et al. 2012a; Fishbain et al. 2012b), violent ideation (Bruns and Disorbio 2000; Bruns et al. 2007), thoughts of killing a physician (Bruns et al. 2010; Fishbain et al. 2009a), thoughts of suing a physician (Fishbain et al. 2007, 2008), borderline personality (Tragesser et al. 2010), medical entitlement (Fishbain et al. 2014c), undefined medical symptoms (Fishbain et al. 2010a), and others.

Both the Block and the Bruns and Disorbio methods have the advantage of being integrated with assessment methods, making them practical for clinical use. For example, the two-tiered approach has been adopted by some evidence-based medicine guidelines for assessing patients with chronic pain (Colorado Division of Workers’ Compensation 2012), and research suggests that this integrated biopsychosocial protocol appears to deliver better care at a reduced cost (Bruns et al. 2012b). However, practical considerations aside, if the standards of evidence-based medicine are strictly applied, the den Boer and Celestin findings have reached a higher standard of evidence.

An advantage of the BHI 2 is that since it was designed to assess the full range of relevant psychological variables, it is able to perform a standardized assessment of nearly all of the variables identified by den Boer/Celestin (Meyer et al. 2012a, 2012b), Block (Disorbio et al. 2012a, 2012b), and Bruns and Disorbio protocols (Bruns and Disorbio 2009). Using the BHI 2, preliminary comparisons of several studies of presurgical psychological evaluation protocols have been published. These studies included the first evidence of the test-retest reliability of presurgical psychological evaluation protocols (Bruns et al. 2012a; Bruns and Disorbio 2009). This information is especially important in forensic evaluations, as the *Daubert* standard of evidence requires that the level of error (i.e., reliability) of a method must be known. The results of these studies showed that using the BHI 2 to assess the den Boer/Celestin, Block, and Bruns and Disorbio protocols led to scores that were highly reliable, with test-retest reliability ranging from .81 to .95. Additionally, all of these presurgical protocols intercorrelated highly, ranging from .63 to .87. The fact that these four divergent methods led to the identification of similar criteria and that the scores intercorrelate strongly is an indication of convergent validity. However, while these four methods produce very similar scores and share half or more of their variance with each other, at the same time, they are all somewhat different. It should be noted that these reliability factors are based on a BHI 2 based assessment, and this would not be applicable to other methods of assessing these protocols.

What can be said about which protocol to use? For a patient who is free of severe psychopathology, the den Boer/Celestin methods are supported by the highest level of evidence. However, these protocols identify only a core set of risk factors that have been well researched. For a more comprehensive assessment of psychopathology, the Block criteria or the Bruns and Disorbio criteria have advantages. The Block protocol has the advantage of having a defined method of clinical implementation, being the approach mostly closely associated with a clinical algorithm, having educational materials for psychologists about surgical procedures, and having a protocol that distills the information into a useful 1 to 5 rating tied to a clinical decision making tree. In contrast, the Bruns and Disorbio protocol differs here by having a somewhat more complex method, but one which generates a standardized score with 1 to 99 percentile ranks based on the BHI 2 norm groups. Suggested cutoffs are above the 84th percentile (1 standard deviation above the mean of patients) for moderate risk, above the 95th percentile for high risk, and above the 99th percentile for very high risk. The Bruns and Disorbio protocol has an important characteristic that it is the only presurgical protocol with evidence that it has no race or gender bias (Bruns and Disorbio 2009). It also has established test-retest reliability for the overall surgical risk estimate, and of all the protocols, it does more to define and identify severe psychopathology. Overall, there are several protocols for assessing psychosocial risk for poor outcome from surgery and other medical treatments, and the BHI 2 is able to provide estimates for all of them. This provides the examiner with the ability to use one test and then afterwards decide which protocol may be most applicable to a particular situation.

Finally, it is worth noting that there is evidence that the core set of psychosocial variables identified in these “presurgical” protocols may also predict the outcome of other medical treatment as well. For example, one study found that presurgical psychological risk factor assessment scores were associated not only with the perception of a good outcome in spinal surgery patients but also with the perception of a good outcome in nonspinal surgery patients, chronic patients, acute pain patients, work hardening patients, patient litigants, and brain injury patients (Bruns and Disorbio 2009). This suggests that these risk factors may apply not only to spinal surgery outcome but also to the outcome of medical treatments generally.

### Pre-opioid Treatment Evaluations

In addition to presurgical psychological evaluations, there are other types of medical treatment where pretreatment psychological evaluations are indicated. One of these is a pretreatment psychological evaluation prior to initiating chronic opioid therapy. Aberrant use of opioids has been observed in up to 24 % of patients (Martell et al. 2007), and the CDC has recently reported that more accidental overdose deaths involve opioid analgesics than heroin and cocaine combined (Centers for Disease Control and Prevention 2012). Not surprisingly, 10 of 13 major opioid guidelines recommend psychological assessment prior to long-term opioid treatment (Nuckols et al. 2013).

Studies suggest that physicians feel conflicted about treating chronic pain with opioids. Surveys of physician attitudes have found that physicians feel that chronic noncancer pain is undertreated, yet at the same time would not prescribe opioids for this condition (Morley-Forster et al. 2003). This reluctance of physicians to prescribe opioids  is associated with two related concerns: opioid craving and opioid addiction. With regard to craving, animal studies have demonstrated that a biological property of opioid medications is that they induce craving (Bai et al. 2014). In human studies, opioid craving has been shown to be associated with opioid relapse in patients who were in treatment for opioid abuse (Tsui et al. 2014) and opioid craving is in turn influenced by both mood (Martel et al. 2014a) and cognitions (Martel et al. 2014b).

A number of risk factors for opioid abuse have been identified in the literature, and almost all of them can be assessed using BHI 2 measures. These include a history of substance abuse (Akbik et al. 2006; Butler et al. 2009; Butler et al. 2008; Edlund et al. 2010; Edlund et al. 2007; Fitzcharles et al. 2011; Green et al. 2009; White et al. 2009), alcoholism (Fitzcharles et al. 2011; Green et al. 2009), drug treatment (Akbik et al. 2006; Butler et al. 2009; Butler et al. 2008), any psychological disorder (Edlund et al. 2010; Edlund et al. 2007; Fitzcharles et al. 2011), depression (Green et al. 2009; Katz et al. 2013; Manchikanti et al. 2007; White et al. 2009; Wilsey et al. 2008), suicidality (Fitzcharles et al. 2011; Green et al. 2009), bad temper (Akbik et al. 2006; Butler et al. 2009; Butler et al. 2008), history of abuse or PTSD (Akbik et al. 2006; Butler et al. 2009; Butler et al. 2008; White et al. 2009; Wilsey et al. 2008), anxiety or panic (Manchikanti et al. 2007; Wilsey et al. 2008), somatization (Manchikanti et al. 2007), and personality disorder (especially borderline or antisocial) (Katz et al. 2013; Kosten et al. 1986; Wilsey et al. 2008). Additionally, social risk factors include tension at home (Akbik et al. 2006; Butler et al. 2009; Butler et al. 2008), conflict with the physician (Akbik et al. 2006; Butler et al. 2009; Butler et al. 2008), younger age (Edlund et al. 2010; Green et al. 2009), low education (Fitzcharles et al. 2011), unemployment (Fitzcharles et al. 2011), and a history of arrest (Akbik et al. 2006; Butler et al. 2009; Butler et al. 2008). A recent study used BHI 2 variables representing the constructs above and used them to predict both the perceived need for pain medication and perceived addiction to pain medicine, identifying the need for medication correctly 73 % of the time, and addiction status correctly 86 % of the time (A. Bruns, Bruns, Disorbio, and Jewell 2014).

Overall, while pre-opioid psychological assessment protocols are not as well developed as presurgical protocols, there is ample evidence of the value of these assessments. With regard to both presurgical and pretreatment evaluations though, the forensic evaluator should be aware that any of the psychological risk scores mentioned here should not be construed as defining the entire evaluation. While these risk scores are important considerations, they should be interpreted within the context of the patient’s history, medical findings, degree of surgical necessity, and other relevant factors.

## Conclusions

The BHI 2 is a biopsychosocial assessment tool whose development was based on a theory of how biological, psychological, and social forces interact in patients with serious injury or illness. While the biopsychosocial model applies to individuals generally, the BHI 2 is especially attuned to the assessment of patients suffering from pain and injury. In the forensic setting, the BHI 2 can be useful in several ways. First of all, a BHI 2 assessment can enable the examiner to perform a biopsychosocial assessment of the patient, which can measure and describe various subjective phenomena, while controlling for patient bias of report. This can provide a deeper understanding of the contributors to the patient’s pain and suffering, by identifying risk factors that appear to be pre-existing, ones that may be reasonably attributed to the onset of a particular medical condition, ones that are consistent with biologic reports, and ones that are difficult to explain biologically.

Like all psychological inventories, the BHI 2 has both strengths and weaknesses. Its strengths include the following:

1. The BHI 2 is a standardized test and was developed based on a biopsychosocial paradigm that is especially well suited for assessing patients with injury, chronic pain, or other somatic symptom disorders.
2. The BHI 2 has both community and patient normative groups, as well as eight other reference groups which can be used to make relevant comparisons to address a variety of clinical questions.
3. The BHI 2 interpretive algorithms integrate community and patient norms into a single continuum, which prevents problems associated with conflicting findings.
4. Consistent with current psychometric concepts, the BHI 2 and BBHI 2 utilize bidirectional scales.
5. The BHI 2 has a short form, the BBHI 2, which can be administered in 10 min or less.
6. The BHI 2 and BBHI 2 are the only instruments that include both a standardized multidimensional measure of pain and validity assessment.
7. The BHI 2 is the only test that can by itself assess all of the presurgical assessment criteria identified by the systematic reviews of den Boer and Celestin. It also has scales that directly or approximately measure the presurgical criteria identified by Block, and by Bruns and Disorbio (excluding determinations that require medical examination or chart review).
8. The BHI 2 has a particular strength in assessing pain, reactions to injury, dysfunctional pain cognitions, and pain-related psychopathology, including somatization, affective distress, substance abuse, characterological traits, suicidality, violent tendencies, and litigious tendencies.

The BHI 2 also has significant weaknesses. They are as follows:

1. In comparison to the MMPI-2-RF, the BHI 2’s validity assessment is not as comprehensive. Although the BHI 2 validity scales were developed through a sound clinical study in a pain and injury population, the MMPI-2-RF has a larger number of validity scales that have been more extensively studied in a variety of populations. The MMPI-2-RF is also able to detect a wider range of psychiatric conditions as this is the test's primary purpose, versus the BHI 2's focus on biopsychosocial assessment.
2. In comparison to the MBMD, the BHI 2 has more of a focus on pain and injury, but less ability to assess how a patient copes with the stress of disease.
3. The independent peer review by the Buros Institute noted above suggested that the greatest need for future BHI 2 research was for further studies on malingering and longitudinal outcome studies.
4. While the validity of many of the BHI 2 scales have been supported by multiple research studies, others have more limited research support.
5. While most of the BHI 2 scales are based on conventional constructs, such as depression and anxiety, others are based on novel constructs, such as symptom dependency, or perseverance. Further research is indicated on these novel scales.
6. A weakness the BHI 2 shares with most inventories is that there have been no studies regarding the long-term reliability of its scales.
7. As noted previously, the BHI 2 approach to assessing depression and anxiety has both strengths and weaknesses. By dividing the assessment of depression into two separate scales, Depression (thoughts and feelings associated with depression) and Vegetative Depression (physical symptoms associated with depression), the BHI 2 controls for false positive depressive findings due to reports of medical symptomatology resembling depression. However, the weakness with this approach is that the full scope of depressive symptomatology is thus spread across two scales, making the assessment of depression somewhat more complex. This approach thus represents a trade-off for the assessment of depression, and the same could be said for the BHI 2 approach to the assessment of anxiety.
  Overall, while the BHI 2 has some identified weaknesses, its numerous strengths pertaining to the assessment of patients with pain or injury will enable it to play a useful role in the forensic psychologist’s toolbox.

## Footnotes

1 Note that the opinion *Cowgar v. Commissioner Of Social Security Administration* was filed with a typo, misspelling “Cowger.”

2 The term “central sensitization” here is derived from current pain theory, which posits that as pain becomes chronic, it becomes less closely associated with peripheral sensations. Instead, the central nervous system becomes sensitized to pain perceptions, resulting in diffuse pain symptoms that are associated with affect, arousal, cognition, memory ,and other factors (Apkarian et al. 2009; Gupta et al. 2007; Melzack and Katz 2006; Staud et al. 2007).
